# The Psychometric Properties of the Relaxation/Meditation/Mindfulness (RMM) Tracker t Inventory in an Iranian Population

**DOI:** 10.1155/2021/2998916

**Published:** 2021-12-31

**Authors:** Alireza Malakoutikhah, Mohammad Ali Zakeri, Ahmad Salehi Derakhtanjani, Mahlagha Dehghan

**Affiliations:** ^1^Student Research Committee, School of Nursing and Midwifery, Kerman University of Medical Sciences, Kerman, Iran; ^2^Non-Communicable Diseases Research Center, Rafsanjan University of Medical Sciences, Rafsanjan, Iran; ^3^Social Determinants of Health Research Centre, Rafsanjan University of Medical Sciences, Rafsanjan, Iran; ^4^Nursing Research Center, Kerman University of Medical Sciences, Kerman, Iran

## Abstract

**Background:**

A growing body of research has examined the psychometric properties of popular mindfulness inventories for different population. The present study is aimed at exploring the psychometric properties and factor structure of the Relaxation/Meditation/Mindfulness (RMM) Tracker t Inventory in Iran.

**Method:**

This was a cross-sectional and methodological study that conducted in Kerman, southeast Iran. Fifty, 300, and 163 Iranian adult participants were participated in the pilot, exploratory, and confirmatory phase, respectively. Face, content, and structural validities, Cronbach's alpha, and Omega coefficient were used to validate the Persian scale.

**Results:**

The results showed that the “Persian version of RMM t” had acceptable content and face validities. The Principal Axis Factoring (PAF) with Promax Rotation showed that the P-RMM t has 3 scales of “Mindful Love, Thankfulness, and Transcendence,” “Relaxation,” and “Mindful Deepening” which further confirmed with confirmatory factor analysis. The internal consistency of all three scales was acceptable (Cronbach's alpha coefficients > 80).

**Conclusion:**

The Persian version of RMM Tracker t seems to be a valid and reliable questionnaire to assess the levels of mindfulness in the Iranian general population.

## 1. Introduction

Mindfulness has its origins in contemplative Eastern traditions and is essential to Buddhist teachings and schools of thought [1]. The term “mindfulness” is defined differently by researchers, practitioners, and clinicians, who value various components of the notion more than others [2].

Kabat-Zinn defines mindfulness as “having moment-to-moment awareness and attention, being in the present moment, and simply being a nonjudgmental observer of current thoughts and emotions without acting on them” [3]. Mindfulness is defined as “being aware of the inputs entering one's mind and noticing what is happening.” Mindfulness is simply the act of observing events and experiences without judging or comparing them [4]. Mindfulness is a warm and friendly, accepting and nonjudgmental attitude toward those elements of the mind. Suspending categorical judgments, which typically follow every perception rather quickly, is an essential component of mindfulness [5]. Mindfulness helps people be in the present moment and accept it for what it is [6]. One is mindful if he or she can pay attention to the present moment rather than being preoccupied with thoughts of the past or the future [7]. As a result, a mindful person maintains direct contact with reality and events occurring both internally (intrapsychic) and externally (environmental) [8].

For over two decades, mindfulness attracted a surge of research. Mindfulness-based interventions have been applied to a wide range of medical and psychological conditions and symptoms, as well as positive emotion, coping, purposefulness, emotional exhaustion, anxiety, stress, depression, self-harm, impulsive and aggressive behaviors, and improving the quality of life [3, 8]. In addition, mindfulness has been used in education, business, and sports as a method of improving functioning [9, 10]. Mindfulness-based interventions include Mindfulness-Based Stress Reduction (MBSR; 18, 19), Dialectical Behavioral Therapy (DBT) [11], Acceptance and Commitment Therapy (ACT) [12], Mindfulness-based Cognitive Therapy (MBCT) [13], and Mindfulness Acceptance Commitment (MAC) [14, 15].

Popular mindfulness questionnaires include the Freiburg Mindfulness Inventory (FMI) [16], the Freiburg Mindfulness Inventory Short Form (FMI-SF) [17], the Mindful Attention and Awareness Scale (MAAS) [18], the Kentucky Inventory of Mindfulness (KIMS) [19], the Toronto Mindfulness Scale (TMS) [20], the Five Facet Mindfulness Questionnaire (FFMQ) [21], the Cognitive and Affective Mindfulness Scale (CAMS) [22], the Cognitive and Affective Mindfulness Scale–Revised (CAMS-R) [23], the Solloway Mindfulness Survey [24], the Southampton Mindfulness Questionnaire (SMQ) [25], the Philadelphia Mindfulness Scale (PHLMS) [26], the Langer Mindfulness Scale [27], the Applied Mindfulness Process Scale (AMPS) [28], and the Body Mindfulness Questionnaire (BMQ) [29].

These scales vary in several important ways; some measure mindfulness as a unidimensional or multidimensional construct [29], while others measure it as a trait-like or state-like construct ([7]; Smith, 2019; Smith, 2021). Some scales measure only an individual's mental state, while others include bodily sensations and experiences [30]. As Grossman [30] noticed, the majority of individuals who design mindfulness measures have personal experience with mindfulness meditation techniques. The extent of this experience seems to have an influence on the items in mindfulness scales. Most definitions of mindfulness focus on moment-to-moment awareness and attention and may not include all aspects defined by Eastern philosophers [30]. According to Walach et al. [17], any instrument will measure some aspects of the concept but not all [17]. Thus, evidences emphasize the development of a proper comprehensive valid instrument for assessing mindfulness [18, 31].

Smith (2019, 2021) has observed that all popular mindfulness inventories are “narrow-spectrum” in that they focus on a restricted range of dimensions, typically present-centered focused awareness and nonjudgmental acceptance. His Third-Generation Mindfulness theory (alternatively Relaxation/Meditation/Mindfulness, or “RMM,” theory) is a “broad-spectrum” perspective that proposes 25 RMM (Relaxation/Meditation/Mindfulness) states organized into “5 + 1” dimensions, that is five levels and one separate dimension of positive mindful emotion. Smith's five levels include Mindful Basic Relaxation, Mindful Quiet Focus, Mindful Awakening, Mindful Deepening, and Mindful Transformation/Transcendence. Mindful Transcendent Positive Emotion is a separate dimension defined by positive emotions such as happy, optimistic, trusting, loving, comparing, compassion, and thankful [32–34]. All 25 RMM states have been related to the practice of mindfulness and mindfulness-related disciplines such as yoga, tai chi, and breathing exercises in the greater mindfulness literature, and all have been singularly identified as independent factors in over 31 factor analytic studies [34–36].

Smith's 25 RMM states and “5 + 1” dimensions are tapped in four RMM Tracker inventories. Each was originally designed as a training tool, written and structured to facilitate mastery of mindfulness skills and self-awareness of broad spectrum of RMM states that can emerge in practice. Over the years, RMM inventories have shown research promise [32, 34, 37].

A growing body of research has examined the psychometric properties of popular mindfulness inventories for the Iranian population. These include the Mindfulness Inventory Short Form (FMI-SF) [38], the Mindful Attention and Awareness Scale (MAAS) [39], the Kentucky Inventory of Mindfulness (KIMS) [40], the Five Facet Mindfulness Questionnaire (FFMQ) [41], the Southampton Mindfulness Questionnaire [42], and the Langer Mindfulness Scale [43]. The present study explores the psychometric properties and factor structure of the RMM Tracker t in Iran.

## 2. Materials and Methods

### 2.1. Study Design and Setting

This is a cross-sectional and methodological study that consists of two phases of forward and backward translation and determination of the validity and reliability of the “RMM Tracker t.” The research setting was Kerman, a city in southeast Iran.

### 2.2. Participants, Sampling, and Sample Size

The study population was all residents of city of Kerman. The study sampled all residents of Kerman, who met the following inclusion criteria: (1) age of at least 18 years, (2) reading and writing abilities, and (3) no self-reported psychiatric disorders.

The convenience sampling method was used for the construct and convergent validity phases, while convenience and purposive sampling methods were used for other phases. Each of the four districts of Kerman (according to the municipal division) was considered as a cluster. Then, the research setting was defined as shopping malls, parks, recreational places, and streets. The number of samples for each section was as follows: (1) qualitative face validity: 12 samples of residents of Kerman, (2) quantitative face validity: 12 samples of residents of Kerman, (3) qualitative content validity: 10 samples of experts, (4) quantitative content validity: 10 samples of experts, (5) pilot study (for checking internal consistency before conducting exploratory factor analysis): 50 residents of Kerman, (6) EFA: 300 samples, (7) CFA: 163 samples, and (8) convergent validity and internal consistency: 463 samples. In addition, 16 questionnaires were excluded from the study because of confounding information and missing values. Sampling took place between April 20, 2020, and April 14, 2021.

### 2.3. Measurements

#### 2.3.1. Demographic Characteristics Form

Age, gender, marital status, educational level, occupation, income, prior knowledge of the mindfulness concept (yes/no), and practice of any mindfulness methods (yes/no) are all included on the Demographic Characteristics Form.

#### 2.3.2. Relaxation/Meditation/Mindfulness (RMM) Tracker t

The RMM Tracker t (trait) is a dispositional or trait inventory used to determine how frequently one experiences various RMM states. Each of its 25 items depicts a distinct psychological state associated with the practice of mindfulness and mindfulness-related disciplines such as breathing, yoga, and tai chi. These items are organized into “5 + 1” dimensions:

Level 1: Mindful Basic Relaxation (4 questions)

Level 2: Mindful Quiet Focus (combination of classical Mindfulness Quiet and Focus) (5 questions)

Level 3: Mindful Awakening (4 questions)

Level 4: Mindful Deepening (4 questions)

Level 5: Mindful Transformation/Transcendence (3 questions)

Mindful TPE is an abbreviation for Mindful Transcendent Positive Emotion.

The RMM Tracker t items are graded on a 13-point Likert scale (never/do not understand item = 0, once a year = 3, once a month = 6, once a week = 9, and about every day = 12) [32].

### 2.4. Procedure, Data Collection, and Data Analysis

#### 2.4.1. Forward and Backward Translation of RMM Tracker t

First, two Farsi-language translators translated the RMM Tracker t into Persian, one of whom was familiar with the psychological concepts. Then, another Farsi-language translator combined the translations. In the next phase, two English-language translators backtranslated the Farsi version into English. Given that the Persian version should be semantically, idiomatically, experientially, and conceptually equivalent to the original version, the research team and translators completed the final edits in the Persian version with the author's (JCS) permission.

#### 2.4.2. Face Validity

For qualitative face validity, 10 participants were interviewed face to face, and difficulty levels (difficulty of comprehending words and sentences), relativity (appropriateness and relation of sentences with the inventory dimensions), and ambiguity (probability of misinterpretation of expressions or inaccuracy of word meanings) were examined.

For quantitative face validity, the Item Impact Method was used to determine the importance of each phrase and reduce items. In this method, the proportion of participants who rated the item as significant (frequency in percentage) is multiplied by the mean score of the item's importance.

(1) $$\begin{array}{c} \text{Item Impact Score}=\text{Significance }\left(\text{Mean}\right)*\text{Frequency }\left(\%\right). \end{array}$$

If the impact score is ≥1.5, the phrase is found to be appropriate for further analysis. At this stage, the inventory was provided to 12 samples located throughout the city [44, 45].

#### 2.4.3. Content Validity

The content validity of the inventory was qualitatively evaluated by experts, including nursing faculty members, psychologists, and methodologists. They were asked to provide written comments on the coverage of content, grammar compliance, the use of appropriate phrases, and the placement of the items.

The Content Validity Index (CVI) was used in the quantitative evaluation of content validity. The Content Validity Index was calculated. Experts were asked to determine the CVI by examining each item on a four-point scale (1 = not relevant, 2 = requires major review, 3 = relevant but needs minor review, 4 = completely relevant). The Item-Content Validity Index score (I-CVI) was calculated by dividing the number of experts who agreed with numbers 3 and 4 by the total number of experts. If the I-CVI value is 0.8, the validity will be accepted. In addition, to calculate the Scale-Content Validity Index (S-CVI), the mean scores of I-CVI of all items were calculated. If the S-CVI of the inventory is 0.9 or higher, it is acceptable [44].

#### 2.4.4. Structural Validity

Exploratory and confirmatory factor analyses were conducted. Both Principal Axis Factoring and Maximum Likelihood were used in exploratory factor analysis to extract the factors (structures), and the varimax and Promax Rotation methods were used to rotate the items. The number of factors was determined using the following criteria: eigenvalues > 1, scree plots, and items with loadings of 0.4 or greater on any one factor [46, 47]. Finally, the best method was determined to be Principal Axis Factoring extraction with Promax Rotation.

Confirmatory factor analysis was used to assess the structure of the factors derived from exploratory factor analysis. The model's adequacy was determined using the chi-squared test. CMIN, Goodness-of-Fit Index (GFI), Comparative Fit Index (CFI), Incremental Fit Index (IFI), and Root Mean Squared Error of Approximation are the main indices used to determine the fit of the model (RMSEA). Acceptable fit of the model is indicated by *χ*2/d.f.<3.0, and RMSEA < 0.08. The GFI, CFI, and IFI indices all had values of ≥0.9 [48].

SPSS18 was used to fit the exploratory factor analysis model to the data and AMOS18 to fit the confirmatory factor analysis model.

#### 2.4.5. Reliability

Internal consistency was tested twice, once before construct validity on 50 samples from the target population and once after factor analysis. To interpret the obtained coefficients, we considered values greater than 0.7 to be acceptable reliability [46].

### 2.5. Ethical Consideration

The Ethics Committee of Kerman University of Medical Sciences approved the project (IR.KMU.REC.1398.673). In addition, the researchers obtained Professor Jonathan C. Smith's permission to translate RMM into Persian. After ethical considerations were approved, the researcher presented the participants with a consent form. This form contains information about the study's purpose and objectives, and the confidentiality of the data, as well as the anonymous participants, who may withdraw from the study at any time. The participants also signed informed consent forms.

## 3. Results

### 3.1. Face and Content Validity

According to 12 participants opinions, the items on the list were incomprehensible to people because of the style of writing, i.e., as inventory format. After contacting JCS, the format of the items was changed to question form with his permission. Face validity was tested on 12 more people, and this time, there was no difficulty understanding the questions in general. Only items 4, 5, 16, 17, and 21 had an item impact score of less than 1.5. These items were candidates for removal from the questionnaire; however, after speaking with JCS, he advised that no items be removed or added to the questionnaire. We discussed the content validity with a panel of experts, including 10 faculty members: 1 = PhD in Clinical Psychology; 1 = PhD in Psychiatric Nursing; 2 = PhD in Nursing, Methodologists; 1 = PhD Candidate in Counseling; 1 = PhD in Nursing with a related thesis; 2 = PhD in Nursing; 2 = MSc in Nursing with experience in working on instrument psychometric properties. The S-CVI was calculated to be 0.95 (mostly had a minor revision comment for most of items). Items 4, 5, 7, and 8 received the most criticism for being unclear. They suggested that additional explanations be added to these items, particularly items 4 and 8, like other items. We added some explanation to the mentioned items based on JCS's advice to make them clear.

### 3.2. Pilot Study: Studying Internal Consistency

The RMM Tracker t was provided to 50 individuals to assess internal consistency, and internal consistency was determined by calculating the Cronbach's alpha coefficient. The item-total correlations range from 0.15 to 0.76, with 23 items having a correlation greater than 0.2 (Table 1). In addition, the Cronbach's alpha values for Mindful Basic Relaxation, Mindful Quiet Focus, Mindful Awakening, Mindful Deepening, Mindful Transformation/Transcendence, and Mindful Transcendent Positive Emotion were 0.74, 0.82, 0.76, 0.75, 0.80, and 0.75, respectively.

### 3.3. Structural Validity

#### 3.3.1. Participants' Characteristics

The mean age of the participants (*n* = 463) was 34.37 ± 10.80 years. The majority of the participants were female, married, employed, and had academic education (Table 2).

#### 3.3.2. Exploratory Factor Analysis

There was no missing data in our sample. The mean scores of the items ranged between 6.2 and 7.39, as shown in Table 3. To check structural validity, first, Bartlett's test of sphericity was used to determine whether the sample size was appropriate for a factor analysis and whether the data came from a sample of the normally distributed population. This test showed statistical significance (*χ*2 = 5374.74, df = 300, *P* < 0.001). In addition to Bartlett's test, the KMO measure of sampling adequacy was examined. In this study, the KMO coefficient was 0.96, confirming the factorability of the correlation matrix of the Persian version of RMM (RMM-P). Both ML and PAF with Promax Rotation were used (there were many cross loading items; therefore, we did not use varimax rotation), and a three-factor solution with an eigenvalue > 1 was retrieved (Table 3). The three-factor solution using the PAF extraction method explained 63.24 percent of the data variance. Scree plots demonstrated that a three-factor solution was reasonable.

#### 3.3.3. Confirmatory Factor Analysis

Following the identification of a three-factor solution via EFA, CFA was used to further test the factor model that emerged from EFA. The first-order confirmatory factor analysis models were used. All of the factor loadings were statistically significant (*t* values > 1.96). The *χ*2-associated *P* value was less than the significance level of 0.05. Except for GFI, other fit indices improved after model modification (*χ*2/d.f. = 1.70, RMSEA = 0.065, GFI = 0.86, CFI = 0.94, and IFI = 0.94). Consequently, we were able to confirm the structure resulting from the exploratory factor analysis using the model.

### 3.4. Reliability

The Cronbach's alpha values for the RMM-P factors ranged from 0.80 (factor 3) to 0.93 (factor 1). The item-total correlations for RMM-P ranged from 0.55 (Item 22) to 0.79 (Items 24) (Table 4).

## 4. Discussion

The aim of the present study was to investigate the psychometric properties of the Persian version of the RMM Tracker t and to determine its factor structure in the general population in Kerman, Iran. The results indicate that the RMM-P instrument had acceptable psychometric properties. The results of this study confirmed the results of the earlier published studies on general population.

The analysis of the P-RMM-Tracker t questionnaire and extraction of three factors showed ten questions related to the first factor that had the highest correlation with each other. When the first factor was examined, the highest score was related to item 24 “loving, caring, compassion” (0.90), followed by item 18 “I felt the sense of something greater” (0.89). The first factor can be combined with the three dimensions of Mindful Awakening, Mindful Deepening, and Mindful Transcendent Positive Emotion in Geise et al. and Smith's studies [33, 49].

The content of the first factor refers to Mindful Awakening and mindful transcendence. Mindfulness can be a source of inspiration for spirituality and mindful transcendence. Mindfulness is characterized by the development of skills for maintaining and paying unique attention to a deep or broad focal goal for an extended period of time, with minimal thought, judgment, or effort [32, 33]. Therefore, this factor is referred to as Mindful Love, Thankfulness, and Transcendence in the current study. Mindfulness opens doors to a spiritual dimension that goes beyond words, but defining this dimension is one of the ambiguities that has raised many questions for people in different societies. However, through mindfulness, we can point to spiritual windows and help clean them more deeply [32, 33].

When the dimensions of the RMM-P questionnaire are examined, nine items related to the second factor have the highest correlation with each other. The highest score in the second factor was related to item 10 “unbothered,” followed by item 11 “easy, effortless.” The second factor is a combination of the two dimensions of Mindful Basic Relaxation and Mindful Quiet Focus discussed in Geise et al. and Smith's studies [33, 49]. The content of the second factor refers to Mindful Basic Relaxation and Mindful Quiet Focus. Therefore, this factor is referred to as relaxation in the current study. Mindfulness teaches us that negative emotions may arise, but they are not a permanent part of our personality. It also allows the individual to respond to events with thought rather than reacting unintentionally and thoughtlessly [50]. Individuals who are aware of this can experience mindful relaxation and focus. Mindfulness means that the person's attention is focused on a stimulus or target for a short period of time. When this attention is complete, it leads to the elimination of annoying stimuli, and the person is drawn to the target. This can occur for any approach to RMM. In progressive muscle relaxation, one might sustain focus on detecting subtle sources of tension, the sensation of tensing up, and of releasing tension. Mindfulness is the acceptance of all external stimuli [37].

Mindfulness, on the other hand, can help people strengthen positive emotions by expanding their attention-concentration, which in turn relaxes them. By expanding one's attention-concentration, one can experience different dimensions to increase creativity and flexibility. Positive emotions counteract the negative effects of negative emotions on people's minds and lead to the release of negative emotions. According to Fredrickson et al., positive emotions can be a fundamental mechanism in meditation and, more broadly, the whole RMM [51]. Mindfulness helps people to concentrate their attention, eliminate distractions and mind wandering, and provide an incentive to stay active [32].

The third dimension of the RMM-P questionnaire consists of four questions. The highest score in the third factor was related to item 17 “Going deeper.” This factor is a combination of some of the questions from the original version factors (including questions about the dimensions of Mindful Awakening, Mindful Deepening, and Mindful Transformation/Transcendence). The content of the third factor refers to Mindful Awakening and transformation. When people are awakened and transformed, they realize that instead of engaging in their thoughts, they can observe their thoughts. In other words, when people learn to observe their thoughts, they value them and avoid judging them [35]. Their mindfulness grows, and they experience a state of Mindful Awakening and transformation. Therefore, factor 3 is referred to as Mindful Deepening in the current study.

It is worth noting that two questions in the original version, namely, question 7 “Periods of sustained, continuous focus, absorption” and question 21 “Awe / wonder, deep mystery,” did not have the necessary correlation to be included in the factors in the Iranian version.

Smith's (2019) concept of mindfulness [33], on the other hand, includes a dynamic level of “5 + 1”: Mindful Basic Relaxation, Focus, Mindful Awakening, Mindful Deepening, Mindful Transformation/Transcendence, plus Mindful Transcendent Positive Emotion. However, we only achieved three factors in the RMM-P version. According to the results of the present study, it is not possible to pinpoint the reason for the combination of the factors expressed in Smith's mindfulness [33] in the three factors discovered in the present study. It is noteworthy that positive emotions in each level of RMM states (Relaxation/Meditation/Mindfulness) express different states of people's levels of experience. People's cultures and habits can influence these experiences and findings. In addition, other factors to consider include the effects of technology, people's habits of paying attention to mindful transcendence and meditation, and differences in sampling.

A review of the literature showed that the factor analysis indices of the RMM questionnaire had not been reported in similar studies. Therefore, it is difficult to compare the results of the present study to those of other communities and groups. However, in the study of Foroughi et al. [42], which examined the psychometrics of the Southampton Mindfulness Questionnaire in Iranian society, three factors, including engagement with thoughts, acceptance, and awareness of thoughts, were extracted from the whole scale after performing factor analysis that explained 51.50 percent of the total variance of mindfulness [42]. López-González et al. [52] also examined the psychometrics of the Relaxation-Mindfulness Scale for Adolescents (EREMIND-A) in children and adolescents. In this study, three factors were discovered that explained 78% of the total variance of mindfulness: attention-concentration in the present moment, relaxation (abilities and attitudes), and sensory (awareness/contemplation/interiority) [52]. Both mentioned studies reported less variance than the present study [42, 52]. However, differences in the types of questionnaires, sampling, and the target population should be considered. Further studies in this area could pave the way for the identification and use of the best tools for measuring mindfulness.

The RMM-P factors showed satisfactory internal consistency. However, in the study of [49], the reported Cronbach's alpha for this questionnaire was between 0.57 and 0.80, which is less than in the present study. This study was conducted on undergraduate students and caregivers, while the present study was conducted on the general community. In addition, the lower sample size (150 samples) and lower mean age (31.71 ± 17.82) in the study of Geise et al. could be the reasons for the difference in the results of the two studies that should be considered. Furthermore, in the study of Smith [33], the reported Cronbach's alpha for a group of people who practiced meditation, yoga, and tai chi for this questionnaire was between 0.75 and 0.83, which is less than in the present study, because participants with different types of exercises participated in the study.

### 4.1. Limitations

The present study was conducted on the Iranian population for the first time with the aim of measuring the psychometric indices of the RMM-P version. This is one of the strengths of this study, which has been conducted to provide a useful tool for measuring mindfulness in Iran. However, this questionnaire has been conducted on the general population and should be examined in different groups. It is suggested that future studies look at the various aspects of this questionnaire. Longitudinal research on the effects of mindfulness over time is also required. Although the size of our sample was sufficient for analysis and validation, better and more accurate results could be achieved by employing a bigger and more diverse sample from different geographical parts of the country. As this study was conducted during the outbreak of COVID-19 disease, the various features of the pandemic should also be addressed, and caution should be exercised when generalizing the results.

## 5. Conclusion

The RMM-P version seems to be a valid and reliable questionnaire to assess the levels of mindfulness in the Iranian general population. The good psychometric properties and strong reliability of the RMM-P questionnaire support its use as a useful tool for assessing the level of mindfulness in multiple dimensions and may provide useful information for managers and researchers working on mental health promotion programs. However, further research is needed to determine the validity of this questionnaire on individuals and other groups in the community.

## Figures and Tables

**Table 1:** Face and content validities and internal consistency of the Persian version of the Relaxation/Meditation/Mindfulness (RMM) Tracker t Inventory.

Item	Face validity (item impact) (*n* = 12)	Content Validity Index (*n* = 10)	Corrected item-total correlation (*n* = 50)
1. Far away	3.41	1	0.15
2. Physically relaxed	2.87	1	0.36
3. At ease, at peace	4.05	0.9	0.54
4. Refreshed	1.44	1	0.60
5. Pleasant mind wandering	1.03	0.8	0.57
6. Lost in fantasy and daydreaming	1.84	1	0.18
7. Periods of sustained, continuous focus, absorption	1.91	0.9	0.49
8. Centered, grounded	1.74	1	0.55
9. Quiet	4.05	1	0.64
10. Unbothered	3.4	1	0.60
11. Easy, effortless	1.78	0.9	0.69
12. Clear, awake, aware	2.87	0.9	0.57
13. Interested, curious, fascinated	1.75	1	0.67
14. Things seemed beautiful	3.19	1	0.70
15. I felt like an observer standing aside and watching what happened	2.09	0.9	0.27
16. Going deeper	1.48	1	0.69
17. Sense of spaciousness, expansiveness	1.07	0.9	0.52
18. I felt the sense of something greater	3.75	1	0.50
19. A sense of meaning, purpose, direction	3.68	1	0.76
20. Feeling reverent, prayerful	2.67	1	0.56
21. Awe/wonder, deep mystery	1.41	1	0.48
22. I felt a profound personal meaningful “spiritual” or “mystical” experience	2.87	0.9	0.56
23. Happy, optimistic, trusting	4.33	0.9	0.74
24. Loving, caring, compassion	4.09	0.9	0.50
25. Thankful. Grateful	4.37	0.9	0.50

**Table 2:** Socio-demographic characteristics of the participants.

Variables	For EFA (*n* = 300)	For CFA (*n* = 163)
	Mean (SD)	Mean (SD)
Age (yr.)	34.58 (11.42)	34.0 (9.57)
	*N* (%)	*N* (%)
Gender		
Male	105 (35.0)	76 (46.6)
Female	195 (65.0)	87 (53.4)
Marital status		
Married	177 (59.0)	106 (65.0)
Unmarried	96 (32.0)	47 (28.8)
Other	27 (9.0)	10 (6.2)
Educational level		
< Diploma	17 (5.7)	6 (3.7)
Diploma	94 (31.3)	56 (34.3)
BSc	145 (48.3)	93 (57.1)
> BSc	44 (14.7)	8 (4.9)
Employment status		
Employed	182 (60.7)	116 (71.2)
Unemployed	102 (34.0)	46 (28.2)
Retired	16 (5.3)	1 (0.6)
Income (million toman)		
<1	90 (30.0)	44 (27.0)
1-2	12 (4.0)	3 (1.8)
2-3	23 (7.7)	15 (9.2)
3-4	38 (12.7)	19 (11.7)
4-5	39 (13.0)	24 (14.7)
5-6	37 (12.3)	26 (16.0)
>6	61 (20.3)	32 (19.6)
Knowledge about mindfulness		
Yes	51 (17.0)	0
No	249 (83.0)	163 (100)
Using mindfulness methods		
Yes	15 (5.0)	0
No	285 (95.0)	163 (100)

EFA: exploratory factor analysis; CFA: confirmatory factor analysis; SD: standard deviation.

**Table 3:** Data description and rotated factor matrix of the Persian version of the Relaxation/Meditation/Mindfulness (RMM) Tracker t Inventory (*n* = 300).

Item	Mean (SD)	Principal Axis Factoring (Promax Rotation)		
		Factor 1	Factor 2	Factor 3
6. Lost in fantasy and daydreaming	7.39 (2.85)	0.47		
12. Clear, awake, aware	7.01 (2.70)	0.57		
13. Interested, curious, fascinated	6.96 (2.85)	0.44		
14. Things seemed beautiful	6.91 (2.65)	0.49		
18. I felt the sense of something greater	7.24 (2.86)	0.89		
19. A sense of meaning, purpose, direction	6.89 (2.82)	0.47		
20. Feeling reverent, prayerful	7.30 (2.77)	0.74		
23. Happy, optimistic, trusting	7.02 (2.74)	0.49		
24. Loving, caring, compassion	7.34 (2.86)	0.90		
25. Thankful. Grateful	7.32 (2.70)	0.86		
1. Far away	6.63 (2.89)		0.58	
2. Physically relaxed	7.28 (2.76)		0.56	
3. At ease, at peace	7.04 (2.82)		0.63	
4. Refreshed	7.02 (2.66)		0.70	
5. Pleasant mind wandering	6.86 (2.69)		0.53	
8. Centered, grounded	7.17 (2.76)		0.46	
9. Quiet	6.64 (2.62)		0.72	
10. Unbothered	6.91 (2.75)		0.80	
11. Easy, effortless	6.71 (2.51)		0.76	
15. I felt like an observer standing aside and watching what happened	6.76 (2.71)			0.56
16. Going deeper	6.65 (2.79)			0.70
17. Sense of spaciousness, expansiveness	6.2 (2.94)			0.57
22. I felt a profound personal meaningful “spiritual” or “mystical” experience	6.53 (2.82)			0.54
7. Periods of sustained, continuous focus, absorption	6.90 (2.67)	—	—	—
21. Awe/wonder, deep mystery	6.85 (2.78)	—	—	—
Eigenvalue		13.16	1.47	1.19
Percentage of explained variance		52.63	5.86	4.75
Cumulative percentage of explained variance		63.24		

SD: standard deviation.

**Table 4:** The internal consistency of the Persian version of the Relaxation/Meditation/Mindfulness (RMM) Tracker t Inventory.

Item	Corrected item-total correlation (*n* = 463)	Cronbach's alpha if item deleted (*n* = 463)
6. Lost in fantasy and daydreaming	0.60	0.93
12. Clear, awake, aware	0.74	0.92
13. Interested, curious, fascinated	0.71	0.92
14. Things seemed beautiful	0.74	0.92
18. I felt the sense of something greater	0.71	0.92
19. A sense of meaning, purpose, direction	0.68	0.92
20. Feeling reverent, prayerful	0.75	0.92
23. Happy, optimistic, trusting	0.75	0.92
24. Loving, caring, compassion	0.79	0.92
25. Thankful. Grateful	0.72	0.92
Factor 1: Mindful Love, Thankfulness, and Transcendence	0.93	
1. Far away	0.54	0.91
2. Physically relaxed	0.69	0.90
3. At ease, at peace	0.76	0.90
4. Refreshed	0.75	0.90
5. Pleasant mind wandering	0.70	0.90
8. Centered, grounded	0.66	0.90
9. Quiet	0.72	0.90
10. Unbothered	0.72	0.90
11. Easy, effortless	0.75	0.90
Factor 2: Relaxation	0.91	
15. I felt like an observer standing aside and watching what happened	0.57	0.78
16. Going deeper	0.73	0.70
17. Sense of spaciousness, expansiveness	0.63	0.75
22. I felt a profound personal meaningful “spiritual” or “mystical” experience	0.55	0.79
Factor 3: Mindful Deepening	0.80	

## Data Availability

Data are available by contacting with the corresponding author by email.

## Additional Points

*Practice Implications*.
